# Draft Genome Sequence of *Marinobacter vinifirmus* Type Strain FB1

**DOI:** 10.1128/genomeA.01058-17

**Published:** 2017-09-28

**Authors:** Victor G. Stepanov, Deborah J. Roberts, George E. Fox

**Affiliations:** a Department of Biology and Biochemistry, University of Houston, Houston, Texas, USA; b School of Engineering, University of British Columbia, Kelowna, British Columbia, Canada

## Abstract

The gammaproteobacterium *Marinobacter vinifirmus* is associated with moderately saline environments and is often found in marine ecosystems. Here, we report the draft genome sequence of *M. vinifirmus* type strain FB1 (3.8 Mbp, 3,588 predicted genes). The presented sequence will improve our understanding of the taxonomy and evolution of the genus *Marinobacter*.

## GENOME ANNOUNCEMENT

*Marinobacter vinifirmus* is a Gram-negative, aerobic, moderately halophilic gammaproteobacterium. It was first isolated from a wine factory wastewater evaporation pond containing tartrate-laden effluents (1). The isolate became the type strain of *Marinobacter vinifirmus* species under the strain name FB1. Since its description by Liebgott et al. in 2006 (1), the species has been detected in various parts of the world’s oceans, which points to its wide distribution around the globe (2–7). However, reliable identification of *M. vinifirmus* strains is hampered by a scarcity of genomic sequence data. The type strain is represented by a single incomplete 16S rRNA gene sequence (GenBank accession number DQ235263), which makes it difficult to unambiguously relate new isolates to the *M. vinifirmus* species. All known *M. vinifirmus* strains are characterized only by their physiological traits and partial 16S rRNA sequences.

Here, we report the draft genome sequence of *M. vinifirmus* type strain FB1. The strain was acquired from DSMZ (Braunschweig, Germany) as “*Marinobacter vinifirmus* DSM 17747.” The cells were cultured in marine broth 2216 (8) at 25°C. Genomic DNA was isolated using the Joint Genome Institute (JGI) cetyltrimethylammonium bromide (CTAB) protocol (http://jgi.doe.gov/user-program-info/pmo-overview/protocols-sample-preparation-information/) and converted to two shotgun libraries with mean insert sizes of 300 bp and 850 bp, respectively, using the Illumina TruSeq DNA PCR-free sample preparation kit LT (Illumina, San Diego, CA). Paired-end sequencing (2 × 150 cycles) was performed on the Illumina NextSeq 500 platform at the University of Houston Seq-N-Edit Core (Houston, TX). The collected sequencing reads were quality filtered and trimmed using Trimmomatic 0.36 (9). Contaminating sequences were removed using DeconSeq 0.4.3 (10). The processed data set was composed of 595,018 read pairs with 311 ± 152-bp inserts, 230,716 read pairs with 841 ± 63-bp inserts, and 267,059 singletons, with a total number of bases in all reads of 239,929,351. The reads were assembled using ABySS 2.0.2 (11), SPAdes 3.9.0 (12), and Ray 2.3.1 (13), and consensus polishing was performed using Mugsy 1.2.3 (14). The assembly yielded 60 contigs of 201 to 520,919 bp in length, with a total length of 3,836,576 bp at 62× coverage and with an *N*_50_ value of 285,212 bp. The average genomic G+C content is 57.99%.

The contigs were annotated using the NCBI Prokaryotic Genome Annotation Pipeline (15). The genome is predicted to contain 3,475 protein-coding genes, 61 RNA-coding genes (of those, 12 rRNA genes in 4 rRNA operons and 45 tRNA genes), and 52 pseudogenes. All four recovered 16S rRNA gene sequences are identical and have four mismatches each with the partial *M. vinifirmus* FB1 16S rRNA gene sequence deposited in 2006 at GenBank (accession number DQ235263) and used since then for taxonomic assignments. A reevaluation of *M. vinifirmus* FB1 taxonomic relationships with other members of the genus *Marinobacter* using the corrected full-length 16S rRNA sequence pointed to two unassigned strains with sequenced genomes, a perchlorate reducer P4B1 (16) and an oil degrader ES-1 (17), as likely belonging to the *M. vinifirmus* species. This finding corroborates with *in silico* DNA-DNA hybridization simulations (18), which showed 74.4% similarity between the FB1 and P4B1 genomes and 70.7% similarity between the FB1 and ES-1 genomes.

### Accession number(s).

The whole-genome shotgun project has been deposited in DDBJ/EMBL/GenBank under accession number NEFY00000000. The version described in this paper is the first version, NEFY01000000.
